# Improving the Characterization of Radiologically Isolated Syndrome Suggestive of Multiple Sclerosis

**DOI:** 10.1371/journal.pone.0019452

**Published:** 2011-04-29

**Authors:** Nicola De Stefano, Maria Laura Stromillo, Francesca Rossi, Marco Battaglini, Antonio Giorgio, Emilio Portaccio, Bahia Hakiki, Gianmichele Malentacchi, Claudio Gasperini, Mario Santangelo, Maria Letizia Bartolozzi, Maria Pia Sormani, Antonio Federico, Maria Pia Amato

**Affiliations:** 1 Department of Neurological and Behavioral Sciences, University of Siena, Siena, Italy; 2 Department of Neurology, University of Florence, Florence, Italy; 3 Neurology Unit, Arezzo Hospital, Arezzo, Italy; 4 Neurology Unit, San Camillo-Forlanini Hospital, Rome, Italy; 5 Neurology Unit, Carpi Hospital, Carpi, Italy; 6 Neurology Unit, Empoli Hospital, Empoli, Italy; 7 Biostatistics Unit, Department of Health Sciences, University of Genoa, Genoa, Italy; Julius-Maximilians-Universität Würzburg, Germany

## Abstract

**Objective:**

To improve the characterization of asymptomatic subjects with brain magnetic resonance imaging (MRI) abnormalities highly suggestive of multiple sclerosis (MS), a condition named as “radiologically isolated syndrome” (RIS).

**Methods:**

Quantitative MRI metrics such as brain volumes and magnetization transfer (MT) were assessed in 19 subjects previously classified as RIS, 20 demographically-matched relapsing-remitting MS (RRMS) patients and 20 healthy controls (HC). Specific measures were: white matter (WM) lesion volumes (LV), total and regional brain volumes, and MT ratio (MTr) in lesions, normal-appearing WM (NAWM) and cortex.

**Results:**

LV was similar in RIS and RRMS, without differences in distribution and frequency at lesion mapping. Brain volumes were similarly lower in RRMS and RIS than in HC (p<0.001). Lesional-MTr was lower in RRMS than in RIS (p = 0.048); NAWM-MTr and cortical-MTr were similar in RIS and HC and lower (p<0.01) in RRMS. These values were particularly lower in RRMS than in RIS in the sensorimotor and memory networks. A multivariate logistic regression analysis showed that 13/19 RIS had ≥70% probability of being classified as RRMS on the basis of their brain volume and lesional-MTr values.

**Conclusions:**

Macroscopic brain damage was similar in RIS and RRMS. However, the subtle tissue damage detected by MTr was milder in RIS than in RRMS in clinically relevant brain regions, suggesting an explanation for the lack of clinical manifestations of subjects with RIS. This new approach could be useful for narrowing down the RIS individuals with a high risk of progression to MS.

## Introduction

Over the past decade, the increasing use of magnetic resonance imaging (MRI) in the diagnostic work-up of pathological conditions has contributed to the uncovering of asymptomatic brain pathologies [1], [2]. Incidental MRI findings are relatively common in the brains of asymptomatic subjects, increasing with age and the use of high-resolution MRI sequences [1].

Recently, a number of studies have specifically focused on subjects who reveal unanticipated brain spatial dissemination of MRI lesions highly suggestive of multiple sclerosis (MS) in the absence of a clinical scenario [3], [4], [5], [6], a condition named as “radiologically isolated syndrome” (RIS) [5].

Much effort has been devoted to attempting to correctly identify and predict the clinical evolution of RIS subjects, in view of the growing consensus for an early disease-modifying treatment in patients diagnosed with MS [7], [8]. Two recent works suggest that the presence of contrast-enhancing and spinal cord lesions increase the risk of disease progression [5], [6]. Currently, however, the natural course of RIS is largely unknown, with the impossibility of clarifying whether the observed changes reflect the earliest form of MS or something else, including a non-disabling form of MS [9].

In line with this, it would be very important to have a better characterization of the brain damage as detected by MRI, occurring in these subjects, thus possibly providing potential predictors of disease progression.

Quantitative MR indices of brain tissue damage such as brain volumes, magnetization transfer (MT) imaging or other pathologically specific MR measures have been largely used in MS and in several other neurological disorders at their early clinical stages and even in the absence of relevant clinical symptoms [10], [11], [12]. Since these MR indices can ensure an accurate assessment of the damage occurring in both lesions and normal-appearing brain regions [13], we measured them in RIS subjects as well as in healthy controls (HC) and relapsing-remitting (RR) MS patients with the aims of *i)* providing a tissue-specific characterization of the brain damage occurring in RIS, *ii)* assessing whether their quantitative brain tissue measures share differences or similarities with those of HC and RRMS patients, and *iii)* determining whether one or more of these measures can be helpful for a better classification of these asymptomatic subjects.

## Methods

### Study population

Nineteen asymptomatic subjects (14 females, 5 males, age: mean 37 years, range 19–51 years) were enrolled in the study. All fulfilled the recently identified criteria for RIS [5], which imply that none of the subjects had previously experienced remitting clinical symptoms consistent with neurologic dysfunction of the central nervous system (CNS). They were all consecutively contacted by the same neurologist (MLS) who followed an identical procedure to avoid potential sampling bias. Table 1 summarizes the demographic and clinical characteristics of the subjects, including the reason for the first MRI, which was performed a mean of 2.41 (range 0.10–6.96) years before. At that time, 15/19 RIS subjects had also performed cervical spine MRI and CSF assessment (Table 1). All subjects agreed to participate in the study, which included an MR examination (without injection of contrast agent) and a full neurological examination, which was independently performed by two raters (MLS and FR) to further exclude signs of CNS dysfunction. Sixteen out of 19 subjects also underwent neuropsychological testing through the Rao Brief Repeatable Battery (BRB) [14]. The MR examinations of the RIS subjects were compared to those of 20 demographically-matched patients with a diagnosis of RRMS according to revised McDonald's criteria [15] (13 females, 7 males, age: mean 37 years, range 24–50 years) and HC (12 females, 8 males, age: mean 37 years, range 22–51 years). To be included in the study, RRMS patients had to be free from relapse and steroid treatment for at least 3 months. They had a relatively early disease stage (disease duration: mean 4.1 years, range 1.0–7.3 years) and mild disability (Expanded Disability Status Scale [EDSS]: median 1.5, range 1.0–5.0). The HC were recruited from laboratory and hospital workers and were included in the group if they had normal neurological examination and no history of neurological disorders.

**Table 1:** Demographic and clinical characteristics of subjects with RIS.

*Subjects*	*Sex*	*Age*	*Family historyof MS*	*Age at first brain MRI*	*Reason for first brain MRI*	*Spinal cord MRI* ∧	*DIT at brain MRI*	*CSF* *	*VEPs* #	*Cognition* $
1	M	45	No	44	Depression	−	−	−	−	+
2	F	51	No	50	Cervical Trauma	+	+	+	+	n.p.
3	M	37	No	35	Dermatitis	−	−	−	−	−
4	F	37	No	34	Headache	n.p.	−	+	−	−
5	M	43	Yes	42	Neuropathic pain	−	+	−	−	n.p.
6	F	46	Yes	40	MS family history	+	−	n.p.	−	+
7	F	23	No	21	Pituitary adenoma	+	+	+	n.p.	−
8	F	45	No	39	Cervical trauma	+	+	+	−	+
9	M	31	Yes	29	Headache	−	+	−	n.p.	−
10	F	38	No	31	Migraine with aura	−	+	+	−	−
11	F	23	No	22	Migraine without aura	+	+	+	+	−
12	M	46	No	45	Headache	n.p.	−	n.p.	−	−
13	F	28	No	23	Facial trauma	−	−	n.p.	n.p.	+
14	F	39	Yes	39	Otosclerosis	n.p.	−	n.p.	n.p.	−
15	F	19	No	19	Headache	+	+	+	+	−
16	F	41	No	40	Dizziness (<2 mins)	−	−	−	−	−
17	F	43	No	43	Anxiety	n.p.	−	+	−	−
18	F	34	No	34	Neck pain	+	+	+	−	−
19	F	31	No	28	Obesity	−	+	+	−	n.p.

n.p. =  not performed.

DIT =  dissemination in time.

CSF =  cerebrospinal fluid.

See text for other abbreviations.

∧ +: presence of cervical spine lesion; -: absence of signal abnormalities.

*+: presence of oligoclonal bands and/or abnormal IgG Index; -: normal pattern.

# +: presence of abnormal P100 in at least one of the eyes; -: normal pattern.

$ +: failure of ≥2 tests at the Rao Brief Repeatable Battery; -: failure of ≤1 test.

The study received approval from the Ethics Committee of the Faculty of Medicine of the University of Siena and informed written consent was obtained from all study subjects.

### MRI examination

All subjects were examined at the MR center of the University of Siena using an identical MR protocol. Acquisitions of brain MRI were obtained in a single session using a Philips Gyroscan operating at 1.5 T (Philips Medical Systems, Best, The Netherlands). A sagittal survey image was used to identify the anterior commissure (AC) and posterior commissure (PC). A dual-echo, turbo spin-echo sequence (TR/TE1/TE2 = 2075/30/90 ms, 256×256 matrix, 1 signal average, 250 mm field of view [FOV], 50 contiguous 3 mm slices) yielding proton density (PD) and T_2_-weighted (T_2_-W) images was acquired in the axial plane parallel to the AC-PC line. Subsequently, an MT sequence was performed acquiring two axial T_1_-weighted (T_1_-W) gradient echo images, one without and one with MT saturation pulses (TR/TE = 35/10 ms, 256×256 matrix, 1 signal average, 250 mm FOV). This sequence yielded image volumes of 50 slices, 3 mm thick, oriented to exactly match the PD/T_2-_W sequence.

Monthly quality assurance sessions were carried out and no major hardware upgrades were done on the scanner during the time of the study.

### MR data analysis

#### Lesions

 For each RIS and RRMS subject, MR scans were first visually assessed by a single observer (FR) who was unaware of subject identity. Labelling of T_2_-W and T_1_-W lesion volume (LV) was then performed by employing a semiautomated segmentation technique based on user-supervised local thresholding (Jim 4.0, Xinapse System, Leicester, UK). For the T_2_-W LV classification, lesion borders were determined primarily on PD images. Information from T_2-_W and T_1_-W images was also considered. T_1_-W hypointense WM lesions were defined as those lesions with signal intensity between that of the grey matter (GM) and the cerebrospinal fluid (CSF) on T_1_-W scans [16]. The value of both T_2_-W and T_1_-W total brain LV was calculated by multiplying lesion area by slice thickness.

Standard-space T_2_-W and T_1_-W lesion probability maps (LPMs) of the RRMS and RIS groups were created by using FSL tools (part of FSL 4.1- http://www.fmrib.ox.ac.uk/fsl/ [17]) and following these sequential steps, as previously described [18]: *i)* binary lesion masks were produced from individual T_2_-W and T_1_-W lesion contours; *ii)* each patient's T_1_-W image was registered to the MNI152 template using a fully affine transformation (12 parameters) and the resulting transformed images were averaged to obtain a study-specific T_1_-W template; *iii)* each patient's PD image was registered to the corresponding T_1_-W image using a rigid body transformation and trilinear interpolation; *iv)* the transformation parameters were applied to the T_2_-W lesion masks, bringing them into alignment with the individual T_1_-W images; *v)* each patient's T_1_-W image was then registered to the T_1_-W template, using a nonlinear registration, and the resulting transformation parameters were applied to the T_2_-W and T_1_-W lesion masks which had been previously registered onto the individual T_1_-W images, using trilinear interpolation. In order to maintain the volume of the transformed lesion masks as closely as possible to those in the native brain images after the trilinear interpolation, the lesion masks were thresholded using a value of 0.5; two observers independently checked all the co-registered lesions masks; *vi)* T_2_-W and T_1_-W LPMs were generated by averaging the T_2_-W and T_1_-W lesion masks, at each voxel, in standard space. For each map, the resulting voxel intensity indicates the probability of lesion occurrence for that voxel.

#### Brain Volume

Brain parenchyma volumes were measured on T_1_-W images by using the cross-sectional version of the SIENA software, SIENAX [19], [20], part of FSL. SIENAX allows global measures of normalized brain volume (NBV) as well as, after tissue-class segmentation, selective measurements of normalized cortical volume (NCV) and normalized WM volume (NWMV) [21]. Reproducibility tests have resulted in a mean standard error across a group of normal subjects of about 0.5–1% [19]. To avoid tissue misclassification due to WM lesions, the latter were masked out and refilled with intensities matching the surrounding normal-appearing WM (NAWM) before each tissue-class segmentation analysis [22].

#### MT

For the analysis of MT data, we used a fully automated procedure previously described [23]. Briefly, saturated (Sat) images were registered to non-saturated (No-Sat) images [17]. The brain was extracted from both Sat and No-Sat images [17] and MT ratio (MTr) images were then calculated using the formula MTr = 100*(No-Sat – Sat)/No-Sat [24]. Mean values of MTr were assessed for the whole brain (WB-MTr) and for different tissue types, produced by using an automated segmentation method [17] to obtain lesional-MTr, ‘non-adjacent-to-lesions’ NAWM MTr and cortical-MTr [23]. Finally, a voxel-based analysis of MTr across the whole brain was performed as previously described [23], [25]. To test for differences in LPM and WB-MTr between the RIS and RRMS groups (unpaired t-test), we used the *randomise* program within FSL to carry out permutation-based testing. Clusters were formed according to a defined threshold (t>3) and corrected for multiple comparisons (across space) by building up the null distribution of the maximum cluster size (for each permutation). Results were considered significant at the 0.05 level. Anatomical locations of the significant GM and WM clusters of MTr analysis were determined by reference to the Harvard-Oxford atlas and to a diffusion tensor imaging (DTI)-based atlas of human WM anatomy, both integrated into FSLView, also part of FSL.

### General statistics

The unpaired t-test was used for comparing RIS subjects and RRMS patients and for comparing RIS subjects with and without DIT. Differences between RIS, RRMS and HC groups were assessed by using analysis of variance (ANOVA) followed by pairwise post-hoc comparisons using Tukey's HSD procedure to account for multiple comparisons. Data were considered significant at the 0.05 level. In addition, a univariate logistic regression model was used to screen the ability of each MRI volumetric (i.e., NBV, NWMV and NCV) and MTr (i.e, WB-MTr, NAWM-MTr, cortical-MTr and lesional-MTr) variable to separate RRMS patients and HC. The most significant volumetric and MTr variable, as estimated by this univariate analysis on RRMS patients and HC, were included into a penalized multivariate logistic regression model. The probability of each RIS subject to be HC or RRMS was estimated according to the following procedure:

- an individual patient score was calculated using the coefficients estimated by the penalized logistic regression:

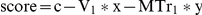
where c was the constant of the model, V_1_ was the most significant volumetric variable and x its estimated coefficient; MTr_1_ was the most significant MTr variable and y its estimated coefficient.
- the probability for a RIS subject to be RRMS was then calculated according to the logistic equation as follows:

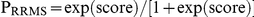

The probability to be HC is 1- P_RRMS_.

The R software version 2.11 was used to perform all the statistical analyses.

## Results

### Clinical and conventional MRI findings

All subjects with RIS had normal neurological examination. At the BRB, 4/16 of the RIS subjects who performed neuropsychological tests showed cognitive impairment (Table1). On conventional MRI, the Barkhof criteria were confirmed in all subjects and, when compared with the previous MRI examination, 10/19 subjects showed at least 1 new T_2_-W lesion, attesting dissemination in time (DIT).

### Load, distribution and frequency of WM lesions

**Figure 1:**
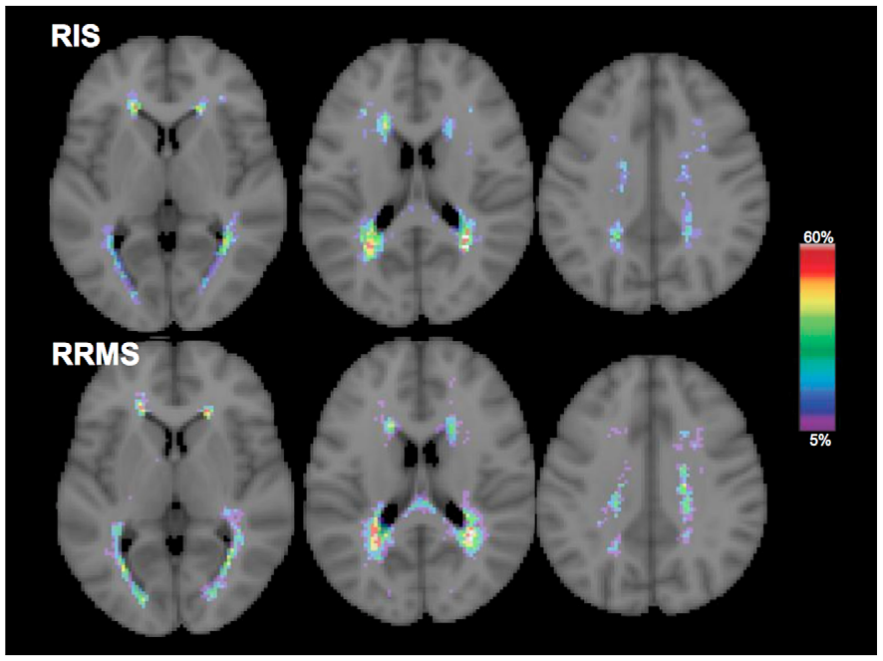
T2-weighted lesion probability maps in RIS and RRMS. The color overlay shows the probability of each voxel containing a lesion in each group. The color bar denotes the probability range. Background image is the MNI standard brain. In both groups, the areas with high probability of containing lesions were similar, mainly involving the superior and posterior regions of the corona radiata, the superior and inferior longitudinal fascicle and the body of the corpus callosum, with similar maximum peaks of lesion occurrence (RIS: 48%, RRMS: 54%). See text for abbreviations.

T_2_-W LV of the RIS group was similar to that of RRMS (RIS: 6.6±6.5 cm^3^, RRMS: 8.4±9.5 cm^3^, p = 0.5). There were no differences in T_1_-W LV between the two groups (RIS: 3.6±4.1 cm^3^, RRMS: 4.5±5.2 cm^3^, p = 0.5). In the RIS group, both T_2_-W and T_1_-W LV were higher in subjects with DIT than in those without DIT (9.3±7.6 cm^3^ versus 3.7±3.5 cm^3^, p = 0.04 for T_2_-W LV; 5.6±4.7 cm^3^ versus 1.5±1.4 cm^3^, p = 0.02 for T_1_-W LV). The LPM analysis (distribution and frequency) of T_2_-W (Figure 1) and T_1_-W lesions did not show differences between RIS and RRMS (p = 0.10).

### Global and tissue-type brain volumes

**Figure 2:**
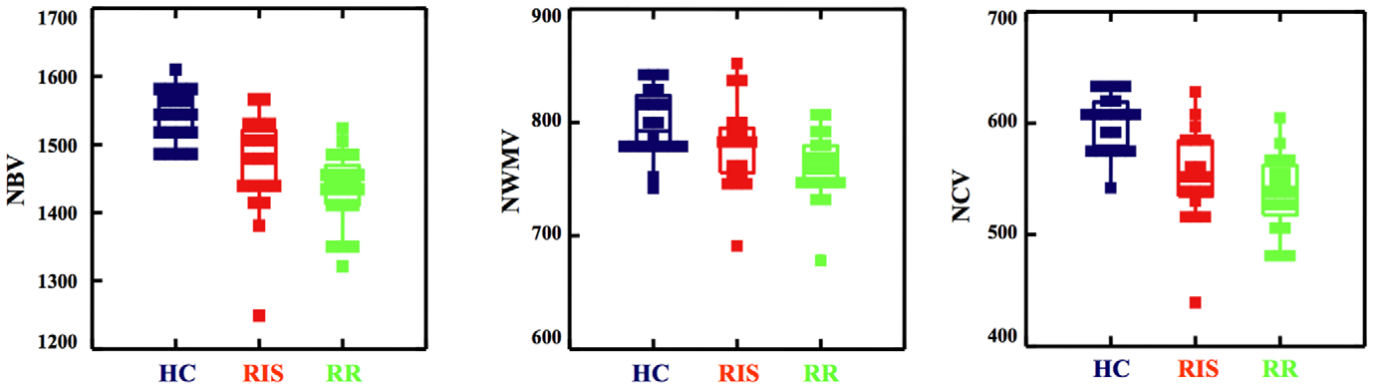
Brain volumes in HC, RIS and RRMS. Box plots comparing the brain volumes of HC (left), RIS subjects (center) and RRMS patients (right). Values are relative to NBV (left), NWMV (center) and NCV (right). Note the presence of similarly low values of brain volume in RIS subjects and RRMS patients with respect to HC. See text for abbreviations.

NBV was similar in RIS and RRMS (1469±75 and 1435±55 cm^3^, p = 0.2), lower in both groups than in HC (1544±36 cm^3^, p<0.0001). NWMV of RIS (779±38 cm^3^) was not different from that of RRMS (761±29 cm^3^, p = 0.25) and HC (799±21 cm^3^, p = 0.16), but this measure was lower in RRMS than in HC (p = 0.002). NCV was comparable in RIS and RRMS (553±42 and 538±33 cm^3^, p = 0.2) and was lower in both groups than in HC (601±24 cm^3^, p<0.0001). All comparisons are shown in Figure 2.

### Global and tissue-type MTr

**Figure 3:**
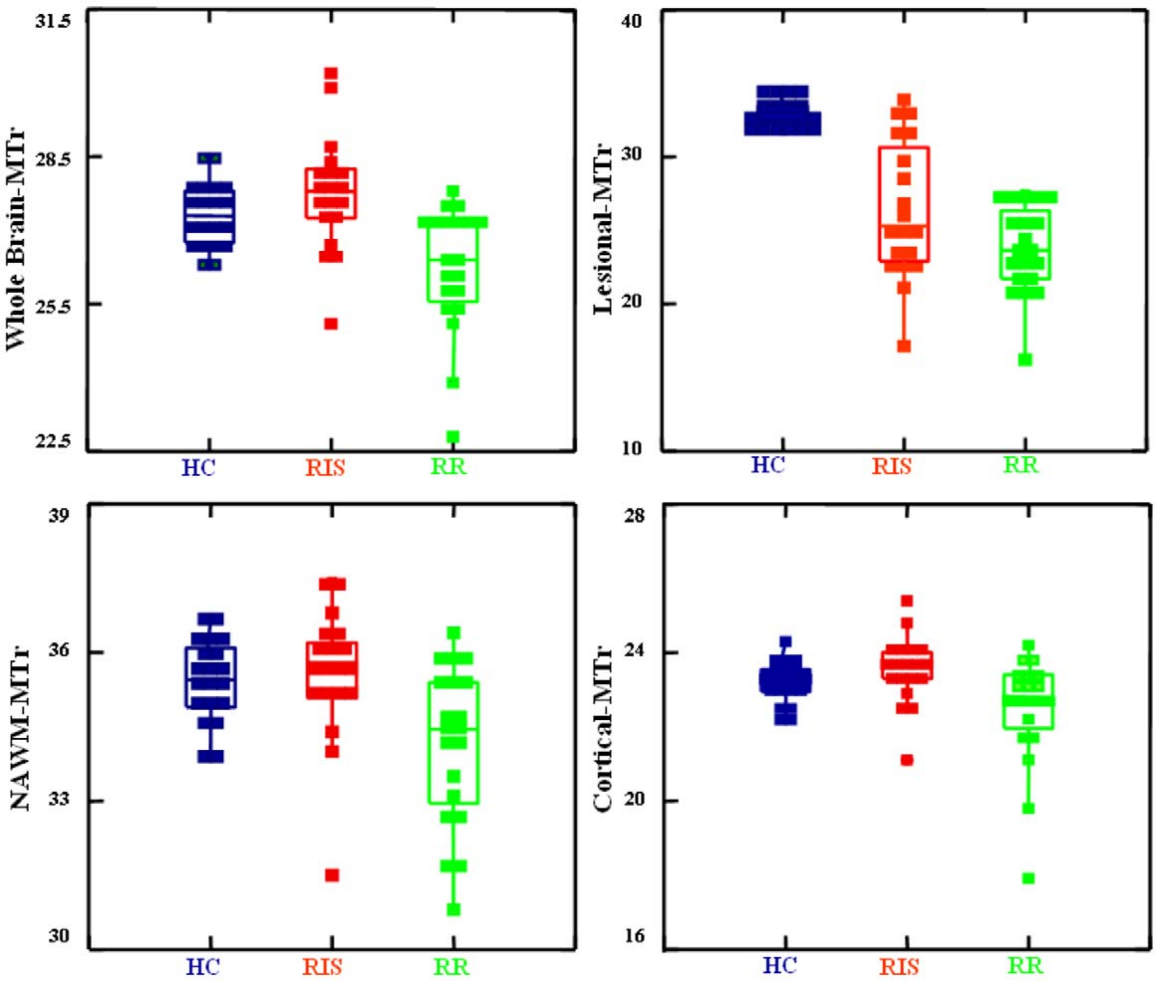
MTr in HC, RIS and RRMS. Box plots comparing the MTr measures of HC (left), RIS subjects (center) and RRMS patients (right). Values are relative to whole brain-MTr (top left), lesional-MTr (top right), NAWM-MTr (bottom left) and cortical-MTr (bottom right). Note the differences between the three groups, with generally higher MTr values in RIS subjects than in RRMS patients. See text for abbreviations.

WB-MTr was similar in RIS and HC (27.8±1 and 27.3±1, p = 0.30) and higher both in RIS and HC than in RRMS (26.2±1, p<0.001 for both). Also, both NAWM-MTr and cortical-MTr values were similar in RIS and HC (NAWM-MTr: 35.6±1 and 35.4±1, p = 0.9; cortical-MTr: 23.5±0.9 and 23.2±0.5, p = 0.6), but were higher in both groups than in RRMS (NAWM-MTr: 34.1±1, p<0.007 for both comparisons; cortical-MTr: 22.5±1.5, p<0.01 for both comparisons). Lesional-MTr values (from T_2_-W lesions) were lower in RIS (26.4±5) and RRMS (23.8±3) than in the homologous brain WM areas of HC (35.4±1; p<0.001), but were higher (p = 0.048) in RIS than in RRMS. All comparisons are shown in Figure 3. In the RIS group, lesional-MTr values were lower in subjects with DIT than in those without DIT (23.7±3.4 versus 29.3±4.3, p = 0.006).

**Figure 4:**
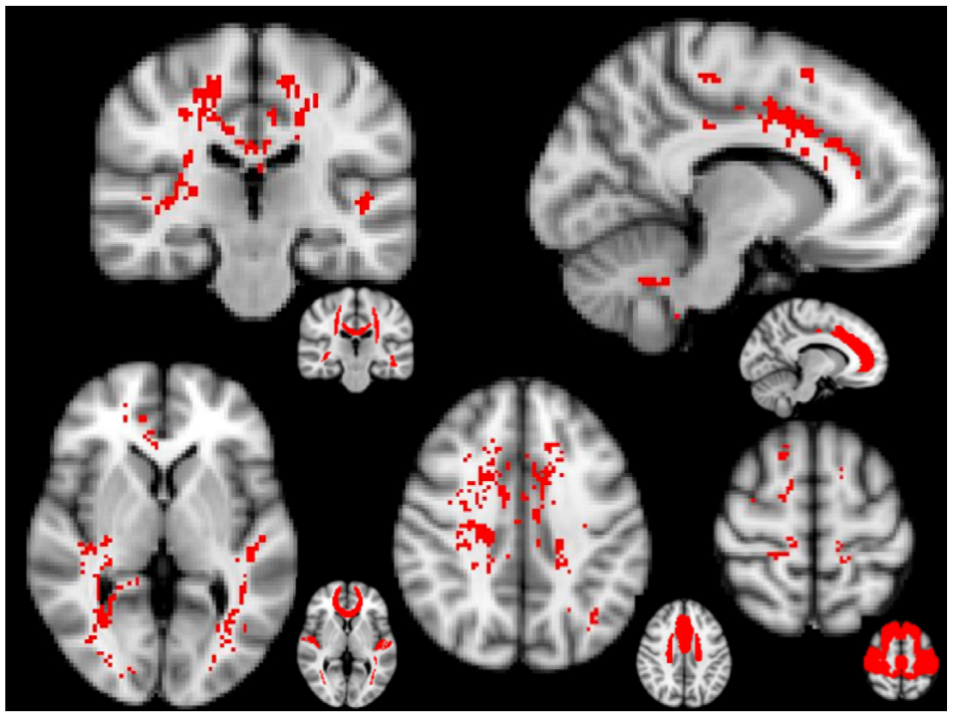
Brain voxelwise MTr comparison between RIS and RRMS. Red voxels show regions with significantly (p<0.05, cluster-corrected for multiple comparisons) lower MTr values in RRMS than in RIS. On smaller brains at the bottom right of each image, red shows the brain regions of the Harvard-Oxford GM atlas and DTI-based WM anatomy atlas corresponding to significant clusters. Background image is the MNI standard brain. Significant brain regions included the superior corona radiata, the body of corpus callosum and the inferior fronto-occipital fascicle (top-left image in coronal orientation); the paracingulate and cingulate gyri (top-right image in sagittal orientation); the inferior longitudinal fascicle, the planum temporale and the genu of corpus callosum (bottom-left image in axial orientation), the superior corona radiata and the paracingulate and cingulate gyri (bottom-center image in axial orientation); and the superior frontal, post- and precentral gyri (bottom-right image in axial orientation). See text for abbreviations.

At the MTr voxelwise analysis, a number of brain areas showed lower MTr values in RRMS than in RIS (p<0.05, cluster-corrected). These included, in the cortex, the bilateral cingulate gyrus and planum temporale, the right pre- and postcentral gyri, superior frontal gyrus and insular cortex and the subcallosal cortex (Figure 4 and Table 2). In the WM, significant regions mainly included the corpus callosum and bilateral areas of the corona radiata and inferior longitudinal/fronto-occipital fascicle (Figure 4 and Table 2).

**Table 2:** Brain regions of the grey and white matter where RIS showed higher MTr than RRMS.

*Cerebral region*	*Side*	*Z-max*	*X*	*Y*	*Z*
*Grey matter*					
Subcallosal cortex	M	5.73	0	12	−8
Superior frontal gyrus	R	5.57	16	18	60
Cingulate gyrus, anterior division	L	5.48	−8	14	34
	R	4.59	12	14	32
	L	4.50	−4	16	22
	L	4.43	−8	−14	42
Cingulate gyrus, posterior division	L	5.40	−6	−26	28
	R	3.99	14	−42	4
Planum temporale	L	5.38	−46	−28	4
	R	3.76	42	−30	10
Paracingulate gyrus	R	4.78	4	44	−2
Lingual gyrus	R	4.57	14	−68	34
Lateral occipital cortex	L	4.14	−34	−68	34
Postcentral gyrus	R	4.08	18	−34	60
Precentral gyrus	R	4.06	34	−8	46
Insular cortex	R	4.04	34	−24	8
*White matter*					
Superior corona radiata	R	5.96	24	0	34
	L	5.08	−12	4	54
Inferior longitudinal fascicle	L	5.52	−46	−28	2
	L	5.05	−34	−78	−2
Body of the corpus callosum	R	5.49	4	−22	24
	R	4.09	2	−10	26
Genu of the corpus callosum	R	5.15	4	26	−2
Inferior fronto-occipital fascicle	R	5.09	24	38	−6
	R	5.04	34	−46	2
Posterior corona radiata	L	4.78	−20	−34	48
Superior cerebellar peduncle	L	4.58	−8	−52	−30
Forceps major	R	4.57	10	−46	12
Middle cerebellar peduncle	L	4.54	−16	−38	−40
Superior longitudinal fascicle	L	4.27	−36	−56	32

Statistically significant regions (p<0.05, cluster-corrected for multiple comparisons) are ordered by decreasing values of the highest local maxima (Z-max). L = left, R = right, M = middle. MNI coordinates are expressed in mm.

### Logistic regression analysis

**Figure 5:**
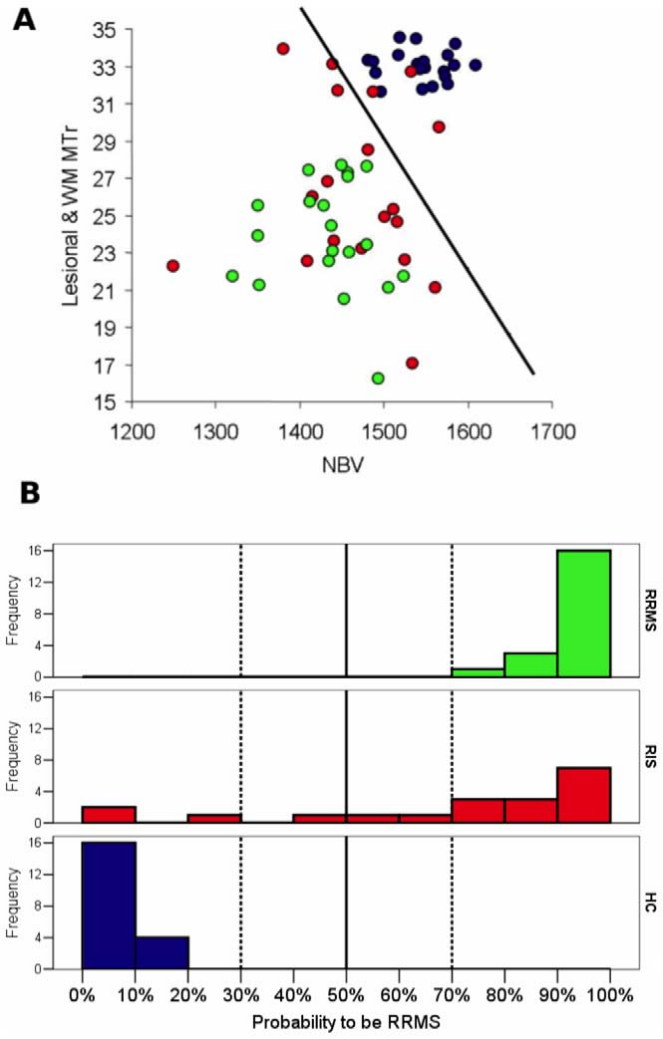
Logistic regression analysis: probability for RIS to be HC or RRMS. Panel A shows the distribution of subjects according to their NBV and lesional-WM MTr. HC are in blu, RIS subjects in red, and RRMS patients in green. Lesional-MTr values were calculated in RIS subjects and RRMS patients from T_2_-W lesional WM regions. MTr values in the WM of HC were calculated from areas homologous to those of the WM lesions of the RIS and RRMS. The black line separates the subjects with a probability of being a RRMS patient below and above 50%, according to the coefficients estimated by the penalized logistic model. The histogram of probability to be RRMS, as estimated according to the penalized logistic model including NBV and lesional-MTr as independent predictors, is reported in panel B. See text for further details.

At the univariate logistic regression analysis all MRI volumetric variables were able to significantly separate RRMS and HC (p ranging from 0.001 to 0.003), with NBV being able to best separate the two groups (accuracy 88%, Nagelkerke R^2^ = 0.84). The MTr variables were also able to separate RRMS and HC (p ranging from 0.001 to 0.04), lesional-MTr values of RRMS being completely separated from the MTr values in homologous WM areas of HC (accuracy = 100%, Nagelkerke R^2^ = 1.0). Thus, NBV and lesional-WM MTr values entered a penalized multivariate logistic regression analysis, to estimate a multiparametric score giving the probability for a RIS subject to be RRMS patient or HC (Figure 5A and B). When the coefficients obtained with the multivariate analysis were applied to the NBV and lesional-WM MTr values of each RIS subject, 13/19 subjects showed a very high probability (>70%; of these, 7/19 subjects had a probability >90%) and 2/19 subjects showed a very low probability (<10%) of being classified as RRMS (and consequently >90% probability of being HC) (Figure 5B).

## Discussion

The unanticipated MRI detection in the brain of asymptomatic subjects of WM lesions suggestive of MS represents a frequent incidental MRI finding, increasing with age [1], in subjects with a history of psychiatric disorders [26] and asymptomatic first-degree relatives of MS patients [27], [28]. Improving the characterization of these MRI findings and establishing whether or not these subjects might be at risk of developing MS could be particularly important as therapy with disease-modifying agents may be more beneficial for MS patients if initiated as early as possible from disease onset [8]. In this context, we used quantitative MR indices to obtain an accurate assessment of the tissue damage occurring in lesions and normal-appearing brain of a group of RIS subjects. By comparing these subjects with demographically-matched HC and RRMS patients, we found that i) focal brain abnormalities, as visualized by conventional MRI, were not different in distribution and frequency between RIS and RRMS; ii) global and tissue-specific (i.e., WM and cortical GM) volumes were similarly lower in RIS and RRMS than in HC; iii) changes in MTr, mostly expression of subtle changes in tissue content and integrity [29], [30], [31] were, in general, higher in RIS subjects than in RRMS patients with, at voxelwise analysis, significant differences in clinically eloquent regions; iv) finally, a multivariate logistic regression analysis allowed for a better characterization of RIS, with as much as 13/19 subjects being classified as RRMS with a probability >70% on the basis of their lesional-MTr and NBV values.

A number of recent studies have assessed conventional MRI in subjects with RIS [3], [4], [5], [6], [32], stressing the similarity of the MRI findings with those of patients with clinically definite MS. In our study, both focal pathology, as expressed by T_2_-W and T_1_-W LV, and diffuse tissue damage, as expressed by global brain volume, were comparable in RIS subjects and RRMS patients. Moreover, the use of LPM allowed to establish that the two groups also showed similar probability of lesion distribution and frequency in a given brain area. Finally, tissue-specific brain volume measurements showed that RIS subjects may have, similarly to RRMS patients, a significant amount of cortical atrophy. Overall, these results suggest that MRI macroscopic damage is similar between RIS and RRMS, with the impossibility of distinguishing the two groups solely on this basis.

MT imaging, which is based on the interactions between free-water protons and protons attached to macromolecules [33], has proven in several studies to be superior to conventional MRI in the detection and quantitation of subtle brain tissue changes [13], [34], [35]. On conventional MRI, protons bound to macromolecules do not directly contribute to the signal, but it is possible to selectively saturate their magnetization with an appropriate off-resonance pulse [33], [36]. This is now routinely obtained from any MR clinical scanner by acquiring two images (with the MT saturation pulse turned on and off, respectively) that are used to generate an MTr image in which the signal intensity of each voxel is determined by the percent MT in that voxel. Thus, when assessed in the brain, MTr measures provide an index of tissue integrity at the cellular level, which in pathological conditions can be an expression of the extent of tissue damage [33], [37], [38]. In such a context, the results of the present study show that the MTr values of RIS subjects were i) similar to those of HC and significantly higher than those of RRMS patients in the normal-appearing brain and ii) much less decreased in WM lesions than those of RRMS patients. Thus, as MTr measures should be able to provide information with considerable pathological specificity [34], [35], and MTr reductions in lesions and normal-appearing brain of MS patients should reflect damage to cellular structures [33], [37], these data strongly suggest that subtle myelin/axonal pathology might effectively be milder in RIS than in RRMS, despite the presence of similar macroscopic brain tissue damage in the two groups. This might be explained by a different degree of demyelination between the two groups, possibly due to a more beneficial response to the demyelinating insult occurring in the RIS subjects. Recent MS studies combining MT imaging and histopathological findings [30], [31], [39] lend support to this interpretation by showing significantly less marked MTr decreases in remyelinated than in demyelinated brain regions. An exceptional capability to repair and/or to withstand the insult leading to demyelination as well as differences in environmental and genetic loads [40] could possibly lead to this peculiar scenario, which is similar to that recently shown in MS patients with benign clinical course [23] and asymptomatic MS relatives with an MRI pattern suggestive of MS [28].

In our study, we also compared the whole brain MTr of RIS subjects and RRMS patients, exploiting the potential of a fully automated voxelwise approach, a powerful tool recently used in several studies on different neurological disorders [23], [41], [42], [43]. Thus, we were able to “clusterize” those WM and GM regions with a significantly higher MTr values in RIS than in RRMS. Interestingly, these brain regions included, in the cortex, areas of the sensorimotor and memory networks and, in the WM, projection, associative, and commissural fibers such as those of the corona radiata, superior and inferior longitudinal fascicle and corpus callosum (see Figure 4 and Table 2). These data indicate clinically eloquent brain regions, usually deeply involved in the pathological substrate of MS, as those that have a minor degree of subtle myelin/axonal pathology in RIS subjects, providing a compelling reason for the lack of clinical manifestations existing in these subjects. It must be stressed here that other factors such as the already mentioned exceptional capability to repair (and reorganize) after an insult could explain why focal demyelination on MRI is not invariably linked to identical clinical manifestations. At any rate, MTr has demonstrated here, as in previous studies [23], [30], [38] to be a very useful and reliable MR metric to (indirectly) assess this *in vivo* [44], [45].

As part of the RIS subjects studied here will become MS patients in the future, a challenge would be to be able to recognize them in advance. Indeed, the clinical relevance of the MR findings can be fully appreciated only with a longitudinal observation of the single RIS subject and, in such a context, previous studies [3], [5] have found that 33% of RIS subjects have their first clinical manifestation after 1–5 year follow-up. However, even a long-term follow-up longitudinal study might not represent the solution to this matter as the disease activity may remain silent for a very long time or even a lifetime [2], [46], [47], [48]. By contrast, in light of providing recommendations on the strategy to follow-up subjects who can potentially develop a disease for which an early treatment is highly recommended, it would be critical to know whether or not subjects with more pronounced brain MR abnormalities will be those who will develop the disease. In recent studies [5], [6], the presence of gadolinium-enhancing lesions on post-contrast T_1_-W images and asymptomatic lesions in the cervical spinal cord, were identified as potential predictors for MRI-based DIT but not for clinical DIT. In our study, we attempted a specific classification of the asymptomatic subjects with RIS with the support of a logistic regression analysis that used quantitative MR indices (i.e., NBV and lesional-WM MTr) to derive a multiparametric score able to provide an estimation of the probability for a RIS subject to be RRMS or HC. On this basis, we found that 13/19 RIS showed a >70% probability of being RRMS. Intriguingly, they included all the 10 subjects who showed abnormal CSF (6 of them with >90% probability of being RRMS), 9/10 subjects who showed a DIT on the new conventional MRI examination and 6/7 RIS subjects with cervical spinal cord lesions, suggesting that this type of analysis, by providing a more specific characterization of the RIS subjects, could potentially narrow down the cohort with a high risk of progression to MS.

In conclusion, the use of quantitative MR markers sensitive to both macroscopic and microscopic pathology provided indications that, while focal and diffuse macroscopic brain damage is by and large similar between RIS and RRMS (and significantly different from HC), the subtle myelin/axonal damage as detected by MTr can be much milder in RIS subjects than in RRMS patients. The evidence that this milder tissue damage occurs in brain regions that are clinically relevant to MS might provide a plausible biological explanation for the lack of clinical manifestations existing in RIS subjects. Finally, we attempted an estimation here of the RIS subjects who were closer to RRMS on the basis of their quantitative MR metrics, providing a characterization of RIS subjects that fully matched with the occurrence of abnormal immunological pattern in these subjects. The clinical relevance of the proposed new approach for a more specific characterization of subjects with RIS needs to be confirmed in future prospective studies able to elucidate whether these and other biomarkers could be successfully used to address the management of subjects with this new pre-clinical condition.
